# In-Traffic Air Pollution Exposure and CC16, Blood Coagulation, and Inflammation Markers in Healthy Adults

**DOI:** 10.1289/ehp.1003151

**Published:** 2011-06-10

**Authors:** Moniek Zuurbier, Gerard Hoek, Marieke Oldenwening, Kees Meliefste, Esmeralda Krop, Peter van den Hazel, Bert Brunekreef

**Affiliations:** 1 Public Health Services Gelderland Midden, Arnhem, the Netherlands; 2 Institute for Risk Assessment Sciences (IRAS), Division of Environmental Epidemiology, and; 3 Julius Center for Health Sciences and Primary Care, Utrecht University, Utrecht, the Netherlands

**Keywords:** air epidemiology, Clara cell protein 16, coagulation, inflammation, interleukins, outdoor air, particulate matter, ultrafine particles

## Abstract

Background: Exposure to traffic-related air pollution is a risk factor for cardiovascular events, probably involving mechanisms of inflammation and coagulation. Little is known about effects of the short exposures encountered while participating in traffic.

Objectives: The objective of the study was to examine effects of exposure of commuters to air pollution on cardiovascular biomarkers.

Methods: Thirty-four healthy adult volunteers commuted for 2 hr by bus, car, or bicycle during the morning rush hour. During the commute, exposure to particle number, particulate matter (PM) ≤ 2.5 µm in aerodynamic diameter (PM_2.5_), PM ≤ 10 µm in diameter (PM_10_), and soot was measured. We estimated inhaled doses based on heart rate monitoring. Shortly before exposure and 6 hr after exposure, blood samples were taken and analyzed for CC16 (Clara cell protein 16), blood cell count, coagulation markers, and inflammation markers. Between June 2007 and June 2008, 352 pre- and postexposure blood samples were collected on 47 test days. We used mixed models to analyze the associations between exposure and changes in health parameters.

Results: We observed no consistent associations between the air pollution exposures and doses and the various biomarkers that we investigated.

Conclusions: Air pollution exposure during commuting was not consistently associated with acute changes in inflammation markers, blood cell counts, or blood coagulation markers.

Exposure to particulate matter (PM) can trigger cardiovascular morbidity and mortality after long-term exposure (years) and short-term exposures (hours to days) (Brook et al. 2010; Burgan et al. 2010; Peters 2005; Vermylen et al. 2005). Possible physiological pathways for cardiovascular effects of air pollution exposure are systemic inflammation, blood coagulation, and changes in the autonomic nervous system (Brook et al. 2010). These mechanisms may eventually lead to myocardial infarctions and arrhythmias, which could lead to hospitalization and mortality (Bhaskaran et al. 2009; Brook et al. 2010; Donaldson et al. 2005). Traffic-related air pollution has been suggested to play an important role (Delfino et al. 2005). Evidence for associations between ambient air pollutants and physiological markers of systemic inflammation and blood coagulation has been obtained in controlled chamber exposure studies and epidemiological studies (Brook et al. 2010). Epidemiological studies have typically assessed the association between ambient air pollution concentrations averaged over the previous 24–72 hr and markers of systemic inflammation and coagulation (Chuang et al. 2007; Hertel et al. 2010; Pekkanen et al. 2000; Peters et al. 2001b; Seaton et al. 1999; Zeka et al. 2006). There is much less information about effects of short (1–2 hr), transient, peak exposures encountered during commuting.

Myocardial infarction was associated with increased participation in traffic in the hours before the onset of the myocardial infarction in a case-crossover study (Peters et al. 2004). The effect was hypothesized to be caused by air pollution exposure in traffic, although alternative explanations such as stress and traffic noise could not be ruled out. Some controlled exposure chamber studies found acute effects of 200–300 µg/m³ diesel exhaust on blood inflammation markers (Mills et al. 2008; Salvi et al. 1999) or coagulation markers (Lucking et al. 2008), but the evidence remains inconsistent (Blomberg et al. 2005; Carlsten et al. 2007; Gong et al. 2008; Mills et al. 2007; Nightingale et al. 2000).

Only a few studies have examined personal, real-life, traffic-related air pollution exposure in relation to acute changes in cardiovascular markers. Riediker et al. (2004)found associations of measured in-vehicle air pollution concentrations with inflammation and coagulation markers in patrol troopers exposed during their approximately 8-hr work shifts. Adar et al. (2007) found a relation between measured in-bus air pollution exposure of a group of elderly subjects and heart rate variability. Jacobs et al. (2010b) found no associations between exposure during 30 min of cycling in traffic and inflammation and coagulation markers, except for a small increase in neutrophils as a percentage of leukocytes.

The Transport-Related Air Pollution, Variance in Commuting, Exposure and Lung Function (TRAVEL) study was designed to examine exposure of commuters to air pollution and associated respiratory and cardiovascular health effects. Details on air pollution exposures encountered during commuting and the related respiratory health effects have been published previously (Zuurbier et al. 2010, 2011). The aim of this study was to assess the acute effects of in-traffic air pollution exposure on several cardiovascular biomarkers.

## Methods

*Study design.* We measured exposure to particle number (PN), particulate matter (PM) ≤ 2.5 µm (PM_2.5_) and ≤ 10 µm (PM_10_) in aerodynamic diameter, and soot during predefined 2-hr morning commutes of 34 healthy, nonsmoking volunteers in cars, buses, and on bicycles. Volunteers were selected through intranet web sites of their employers. The inclusion criteria were age between 18 and 56 years and nonsmoking. We took measurements between 0800 and 1000 hours on 47 weekdays during June 2007 through June 2008. Exposure contrasts derived from day-to-day variation and differences in commuting mode on each sampling day. We collected blood samples before exposure and 6 hr after traffic exposure at the same times each day to keep circadian variation constant; air pollution exposure varied from day to day. We selected blood parameters and time points after reviewing previous studies of ambient and controlled exposures (Gong et al. 2003; Mills et al. 2005, 2007, 2008; Nightingale et al. 2000; Pekkanen et al. 2000; Peters et al. 2001a, 2001b, 2004; Riediker et al. 2004; Salvi et al. 1999; Seaton et al. 1999). Because we studied a range of respiratory, inflammatory, and coagulation markers, we selected a time point for measuring outcomes that was a compromise among the time points at which the individual markers were expected to be most likely to change in response to exposure. Specifically, we collected blood 6 hr after exposure because of *a*) reported increases in neutrophils and platelets in blood 6 hr after a 1-hr experimental exposure of healthy volunteers to fresh diesel exhaust (Salvi et al. 1999); *b*) an increase in platelets 2 hr after exposure to concentrated ambient fine and ultrafine particles (Mills et al. 2008); *c*) an increase in platelet activation and thrombus formation 6 hr after exposure to diesel exhaust (Lucking et al. 2008); and *d*) an increase in C-reactive protein (CRP) immediately after and 4 hr after exposure of healthy volunteers and volunteers with asthma to concentrated ambient ultrafine particles (Gong et al. 2008). In addition, two epidemiological studies by Peters et al. found an association between onset of myocardial infarction and exposure to traffic (Peters et al. 2004) and PM_2.5_ (Peters et al. 2001a) 2–4 hr before the myocardial infarction. Furthermore, several studies documented significant airway inflammation at that time point (Salvi et al. 1999; Stenfors et al. 2004) or at 4 hr postexposure (Nightingale et al. 2000). An important mechanism for systemic inflammation is prior airway inflammation.

We measured exposures during morning rush hour when they were expected to be highest and because exposures would be minimized immediately before the experiment. We selected subjects living close (< 20 min) to the starting point of exposure to limit prior exposures and selected office workers to limit exposures between commuting and the health evaluation 6 hr after exposure. We excluded participants who were exposed to fumes or dust at work.

*Commuting modes. S*ampling days were divided among cycling sampling days (which included two different cycling routes that varied in traffic intensity), car sampling days (including diesel and gasoline-powered cars), and bus sampling days (including both electric trolley buses and diesel buses). All measurements were performed in Arnhem, a medium- sized Dutch city (145,000 inhabitants). On cycling sampling days, technicians rode three-wheeled cargo bicycles to transport the equipment; on car sampling days the cars were driven by the technicians. On each sampling day, three or four volunteers rode in each type of car, bus, or cycle route. The volunteers were scheduled to participate in all commuting modes for a maximum of 12 times.

*Exposure assessment.* The exposure assessment has been reported in detail previously (Zuurbier et al. 2010). We measured PN concentrations using condensation particle counters (CPC; model 3007; TSI Inc., Shoreview, MN, USA). We measured PM_2.5_ with active sampling personal DataRAMs (model 1200; MIE Inc., Bedford, MA, USA). Both CPCs and DataRAMs measured real-time concentrations, recording every second. We collected PM_10_ using Harvard Impactors (Air Diagnostics and Engineering Inc., Naples, ME, USA). We determined soot content of the filters using a smoke stain reflectometer (model M43D; Diffusion Systems Ltd., London, UK) and converted the values into an absorption coefficient. For estimations of elemental carbon (EC) inhaled doses, we converted absorption (soot) levels into EC concentrations using the equation EC (micrograms per cubic meter) = 1.6053 × absorption (10^–5^per meter) – 0.2620 (Cyrys et al. 2003). Hourly ambient nitrogen dioxide (NO_2_), ozone (O_3_), and PM_10_ concentrations measured with chemiluminescence, ultraviolet absorption, and beta attenuation continuous monitors were obtained from the Dutch National Air Quality Monitoring Network [for additional details, see Supplemental Material, p. 2 (http://dx.doi.org/10.1289/ehp.1003151)].

In addition to measuring exposures to air pollutants, we estimated doses of inhaled air pollutants to account for differences in ventilation during cycling compared with commuting by car or bus. Specifically, we estimated minute ventilation levels for all individuals during all trips, based on their heart rate measurements during commuting (measured using Polar RS400 heart rate monitors; Polar Electro, Kempele, Finland) and previously determined information for each individual participant on the relation between their heart rate and minute ventilation (Zuurbier et al. 2009). Inhaled doses were calculated by multiplying concentrations, minute ventilation, and duration of the trip, divided by body surface area (BSA) to correct for differences in airway epithelial surface area. We calculated BSA using the equation


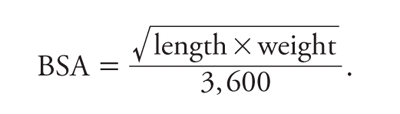


*Clinical measurements.* We collected venous blood before exposure and 6 hr after the end of exposure. Samples were transported to a local hospital laboratory within 1 hr of blood sampling and immediately processed. Blood cell counts were performed immediately. For all other analyses, plasma was stored at –80°C, and all samples were analyzed at the same time after the fieldwork period ended. The study was approved by the medical ethical committee of Utrecht University Medical Centre. All participants gave written informed consent prior to the study.

Inflammation markers. We analyzed CRP using a CRP Vario high-sensitivity assay (Architect, Abbott Diagnostics, Hoofddorp, the Netherlands). Interleukin (IL) 6, IL8, IL10, and tumor-necrosis factor α (TNFα) were analyzed in duplicate, using a Luminex assay (Hosnijeh et al. 2010). We used mean values in analyses.

Marker of lung epithelial damage. We analyzed Clara cell protein 16 (CC16) using a sandwich enzyme immunoassay (CC16 ELISA kit; DiaMed EuroGen, Turnhout, Belgium) as described by the manufacturer.

Blood cell number counts. We performed analyses on white and red blood cell counts and platelets using the automated XE-2100 hematology analyzer (Sysmex Nederland B.V., Etten-Leur, Netherlands).

Blood coagulation markers. We analyzed prothrombin time (PT), activated partial thromboplastin time (APTT), platelets, fibrinogen, factor VII, and von Willebrand factor (vWF) using the STA-R automated coagulation analyzer (Diagnostica Stago, Asnières-sur-Seine, France),

*Statistical analyses.* We used mixed models to analyze relations between variation in either the measured exposures or estimated inhaled doses of each individual air pollutant (PN, PM_2.5_, PM_10_, or EC, in separate models) and the blood parameters. We used repeated measurement analyses to correct for correlation between measurements of the same subject, and we included random intercepts for subjects to account for individual differences.

Changes in biomarkers between postexposure and preexposure measurements were used as dependent variables. We used log transformations of postexposure and preexposure measurements when the distribution of the residuals of post exposure – pre exposure differences were right-skewed. This was the case for CRP, IL6, IL8, IL10, and TNFα. Relatively large numbers of samples were below the detection limit and at the lower end of the calibration curve for IL6, IL8, IL10, and TNFα [see Supplemental Material, p. 4 (http://dx.doi.org/10.1289/ehp.1003151)]; therefore, we also performed logistic regression analyses for these markers, dichotomizing them at the 80th percentiles of the preexposure to postexposure changes.

The magnitude of change in health outcomes was calculated for interquartile range (IQR) increases in exposures or estimated doses. Potential confounders included in models for all end points were relative humidity and temperature (2-hr means, measured during commuting), season (spring, autumn, and winter; there were no measurements in summer), and minutes of traffic participation before baseline measurements and separately for time in traffic between 1000 and 1600 hours. We also included a variable indicating whether commuting was by bicycle or by car/bus in the model, because we observed an independent effect of cycling on several markers in blood. Mean ambient background NO_2_ concentrations during the 24 hr before commuting exposure were included to account for potential effects of ambient air pollution on pre- and postcommuting outcome measurements. Sensitivity analyses were performed using ambient PM_10_ or ambient O_3_ instead of ambient NO_2_ and by excluding observations with cough at baseline as a marker of a cold. Effect modification was analyzed using stratified analyses for cycling versus riding by car/bus, body mass index (BMI; categorized as below or above 25 kg/m²), and fruit and vitamin intake (the high category defined as seven or more fruits per week or taking vitamin supplements on ≥ 5 days/week). To assess the influence of outliers on regressions, we performed additional analyses leaving out 1% (maximum of four) of observations with the highest Cook’s distance values. Level of significance was defined as a *p*-value of 0.05. Data were analyzed using SAS 9.2 (SAS Institute Inc., Cary, NC, USA).

## Results

**Figure 1:**
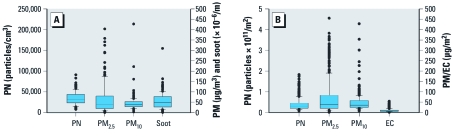
Air pollutant exposures (*A*) and inhaled doses (*B*) in transport. In *A*,* n* = 93, with two commuting modes on 47 sampling days and one mode missing. PM_2.5_ was measured photometrically, whereas PM_10_ was measured gravimetrically. Photometric instruments can overestimate exposures; therefore, PM_2.5_ is high compared with PM_10_. In *B*, *n* = 345, with 34 volunteers participating 5–12 times; doses were adjusted for BSA.

*Exposure.* We performed measurements on 16 bicycle-sampling days, 16 car-sampling days, and 15 bus-sampling days. Exposures to and inhaled doses of all pollutants varied widely between days (Figure 1). PM_2.5_ concentrations were likely overestimated because of the photometrical instrument used (Zuurbier et al. 2010), but the differences in exposures between measurement days are likely to be valid measures of the actual differences. Because of their increased physical activity, the estimated minute ventilation of cyclists was higher than that of car and bus passengers. Estimated minute ventilations of cyclists were 23.5 L/min, on average, compared with 11.8 for car passengers and 12.7 L/min for bus passengers (Zuurbier et al. 2009).

In-traffic exposures were substantially higher than were urban background concentrations measured during rush hour (e.g., for soot, 2.0–3.7 times higher; for PN, 1.6–2.5 times higher) (Zuurbier et al. 2010). Ambient NO_2_ and O_3_ exposure levels of the 24 hr preceding the start of exposure ranged from 15 to 66 µg/m³ (mean, 32 µg/m³) and from 4 to 141 µg/m³ (mean, 56 µg/m³), respectively. Ambient PM_10_ of the preceding day ranged from 13 to 55 µg/m³ (mean, 28 µg/m³).

Spearman correlation coefficients were 0.58, 0.32, and 0.45 between soot and PN, PM_2.5_, and PM_10_ exposures, respectively, and 0.38 between PM_2.5_ and PM_10_ exposures (*p* < 0.01 for all). PN exposure was not correlated with PM_2.5_ or PM_10_ exposures. Estimated doses were correlated among all of the pollutants, with coefficients ranging from 0.34 (for PN and PM_2.5_ doses) to 0.74 (for PN and EC doses) (*p* < 0.01 for all).

*Clinical measurements.* Characteristics of the 34 participants are given in Table 1. In total, 352 individual health outcome measurements were conducted on 47 sampling days (pre- and postexposure). Each subject participated on at least 5 days, with a maximum of 12 days.

**Table 1:** Descriptive characteristics of the subjects (*n* = 34).

Characteristic	Mean (range) or *n* (%)
Age (years)	42.0 (23–55)
BMI (kg/m²^)^	
< 25	19 (56)
≥ 25	15 (44)
BSA (m²)*a*	2.0 (1.5–2.3)
Sex	
Male	24 (71)
Female	10 (29)
Ex-smokers	10 (29)
Fruit and vitamin intake*b*	
High*c*	20 (59)
Low	13 (38)
**a**BSA = √ (length × weight)/3,600. **b**Data were missing for one person. **c**Diet included ≥ 7 fruits per week or taking vitamin supplements on ≥ 5 days/week.	

By specifying random intercepts, we assured assessment of associations between temporal within-person variability of biological markers and air pollution exposure. Mean baseline levels and mean post exposure – preexposure differences of the biomarkers are reported in Supplemental Material, Table 1 (http://dx.doi.org/10.1289/ehp.1003151).

*Associations between air pollution and blood inflammation markers.* We did not find consistent associations of air pollution with changes in IL6, IL8, TNFα, CRP, or CC16 (Table 2). An IQR increase in mean PM_2.5_ exposure was associated with a 5.4% decrease in IL10 [95% confidence interval (CI), –11 to –0.2; *p* = 0.04], contrary to expectations. PN dose was associated with a 14% decrease in TNFα (95% CI, –31 to 2.6; *p* = 0.10). Consistent with our hypothesis, PN dose was positively associated with CRP (1.8%; 95% CI, –0.1 to 3.8; *p* = 0.07) and PN exposure with IL8 (17%; 95% CI, –0.9 to 34; *p* = 0.06). The logistic regression produced no significant associations between air pollution and inflammation markers (data not shown). Estimates that were not adjusted for potential confounders were comparable with the adjusted estimates (data not shown). The confounding effect of cycling was a significant predictor of IL10 and TNFα. In stratified analysis, IL10 was associated with soot exposure during car/bus trips (5.0%; 95% CI, –0.5 to 11; *p* = 0.08) but not bicycle trips (–0.6%; 95% CI, –16 to 14; *p* = 0.94), but differences between strata were not significant [see Supplemental Material, Table 3, (http://dx.doi.org/10.1289/ehp.1003151)]. We observed no significant differences with TNFα between bicycle trips and car/bus trips. An analysis excluding the 26 observations with cough reported at baseline as a marker of a cold did not change the results.

**Table 2:** Effect estimates [% (95% CI)] for differences in markers of inflammation and lung epithelial damage per IQR change in exposure and inhaled dose.

Dose/exposure	IL6	IL8	IL10	TNFα	CRP	CC16
PN dose		–0.8 (–14 to 13)		5.9 (–8.4 to 20)		0.3 (–4.7 to 5.4)		–14 (–31 to 2.6)*		1.8 (–0.1 to 3.8)*		0.2 (–2.7 to 3.0)
PN exposure		–14 (–30 to 3.1)		17 (–0.9 to 34)*		–1.2 (–7.2 to 4.8)		–16 (–37 to 6.3)		0.0 (–2.5 to 2.4)		–1.6 (–4.7 to 1.6)
PM_2.5_ dose		–2.6 (–13 to 8.0)		–3.0 (–14 to 8.2)		–2.8 (–6.5 to 1.0)		–1.8 (–16 to 12.1)		–0.6 (–2.2 to 0.9)		–1.3 (–3.4 to 0.8)
PM_2.5_ exposure		–1.3 (–15 to 13)		–8.3 (–24 to 7.0)		–5.4 (–11 to –0.2)**		–0.6 (–20 to 19)		–1.6 (–3.8 to 0.5)		–1.8 (–4.5 to 1.0)
PM_10_ dose		–3.1 (–18 to 12)		–3.9 (20 to 12)		1.0 (–4.5 to 6.5)		–5.7 (–24 to 12)		0.1 (–2.1 to 2.3)		–2.0 (–5.0 to 1.0)
PM_10_ exposure		–3.1 (–15 to 8.5)		–0.4 (–2.9 to 12)		–0.4 (–4.7 to 3.8)		–1.9 (–17 to 13)		–0.7 (–2.5 to 1.0)		–1.4 (–3.6 to 0.8)
Soot dose (EC)		–0.2 (–11 to 10)		3.2 (–8.2 to 15)		1.4 (–2.5 to 5.3)		–8.9 (–22 to 4.7)		0.9 (–0.7 to 2.5)		–0.9 (–3.0 to 1.2)
Soot exposure		–3.5 (–15 to 8.2)		3.4 (–9.5 to 16)		0.8 (–3.6 to 5.2)		–7.4 (–23 to 8.7)		0.0 (–1.8 to 1.8)		–1.9 (–4.1 to 0.4)
Log-transformed values were used for CRP, IL6, IL8, IL10, and TNFα; for CC16, percent change was calculated as estimate divided by the mean baseline value. Estimates were calculated using mixed-model analyses per IQR change (95% CI). IQRs of 2-hr mean values were as follows: PN, 18,195 pt/cm³ (exposure) and 2.40 × 10^10^ pt/m^2^ (dose); PM_2.5_, 68.1 µg/m³ (exposure) and 61.9 µg/m² (dose); PM_10_, 20.8 µg/m³ (exposure) and 32.4 µg/m² (dose); soot, 3.51 × 10^–5^/m (exposure) and 6.31 µg/m² (EC dose, calculated from soot absorption). Doses were adjusted for BSA; exposures were adjusted for relative humidity, temperature, season, time test was taken, ambient NO_2_, cycling, and time privately spent in traffic before 0800 hours and between 1000 and 1600 hours. *p < 0.10. **p < 0.05.												

**Table 3:** Effect estimates [% (95% CI)] for differences in blood cell counts per IQR change in exposure and inhaled dose.

Dose/exposure	Erythrocytes (%)	Leukocytes (%)	Neutrophils (%)	Lymphocytes (%)
PN dose		0.1 (–0.3 to 0.4)		–0.5 (–2.3 to 1.4)		–2.0 (–5.4 to 1.5)		0.1 (–2.1 to 2.3)
PN exposure		–0.1 (–0.5 to 0.4)		–1.6 (–3.8 to 0.6)		–3.8 (–7.6 to 0.0)*		0.7 (–1.8 to 3.3)
PM_2.5_ dose		–0.2 (–0.5 to 0.1)		–0.8 (–2.2 to 0.5)		–2.5 (–5.0 to –0.1)**		0.7 (–0.8 to 2.3)
PM_2.5_ exposure		–0.3 (–0.7 to 0.1)		–0.7 (–2.6 to 1.2)		–2.4 (–5.7 to 0.8)		1.3 (–0.9 to 3.4)
PM_10_ dose		–0.1 (–0.6 to 0.2)		–0.1 (–2.2 to 1.9)		–0.1 (–3.7 to 3.5)		–1.3 (–3.7 to 1.0)
PM_10_ exposure		–0.2 (–0.5 to 0.1)		–0.7 (–2.2 to 0.9)		–0.7 (–3.4 to 1.9)		–0.9 (–2.6 to 0.9)
Soot dose (EC)		0.1 (–0.1 to 0.4)		–0.7 (–2.2 to 0.7)		–1.5 (–4.1 to 1.1)		–0.3 (–2.0 to 1.3)
Soot exposure		0.1 (–0.2 to 0.4)		–0.9 (–2.5 to 0.7)		–1.6 (–4.4 to 1.2)		0.0 (–1.8 to 1.8)
Change was calculated as the estimate divided by the mean baseline value. Estimates were calculated using mixed-model analyses per IQR change (95% CI). IQRs of 2-hr mean values were as follows: PN, 18,195 pt/cm³ (exposure) and 2.40 × 10^10^ pt/m^2^ (dose); PM_2.5_, 68.1 µg/m³ (exposure) and 61.9 µg/m² (dose); PM_10_, 20.8 µg/m³ (exposure) and 32.4 µg/m² (dose); soot 3.51 × 10^–5^/m (exposure) and 6.31 µg/m² (EC dose, calculated from soot absorption). Doses were adjusted for BSA; exposures were adjusted for relative humidity, temperature, season, time test was taken, ambient NO_2_, cycling, and time privately spent in traffic before 0800 and between 1000 and 1600 hours. *p < 0.10. **p < 0.05.								

*Associations between air pollution and blood cell counts.* We found no associations between IQR increases in mean air pollution levels and erythrocytes, lymphocytes (Table 3), basophils, eosinophils, and monocytes [Supplemental Material, Table 4, (http://dx.doi.org/10.1289/ehp.1003151)]. There were consistent but nonsignificant negative associations of all pollutants and leukocytes (–0.1 to –1.6%), and of PN and PM_2.5_ and neutrophils (–2.0 to –3.8%) (Table 3); these associations were opposite from our hypothesis. The associations between PN exposure and dose and neutrophils became significant and stronger after removal of four influential data points with highest Cook’s distance values (data not shown). Stratified analyses showed stronger negative associations with leukocyte and neutrophil counts in cycling trips than in car/bus trips of PN, PM_2.5_, and soot (not PM_10_). Mean PN exposure during cycling trips was associated with increases of 4.9 and 11% (*p* = 0.08 and *p* = 0.04) in leukocytes and neutrophils, respectively, whereas associations were 1.0 and 0.4% (*p* = 0.48 and *p* = 0.85) for car/bus trips (see Supplemental Material, Table 5).

**Table 4:** Effect estimates [% (95% CI)] for differences in coagulation markers per IQR change in exposure and inhaled dose.

Dose/exposure	APTT (%)	PT (%)	vWF (%)	Factor VII (%)	Platelets (%)	Fibrinogen (%)
PN dose		0.0 (–0.4 to 0.3)		0.3 (–0.1 to 0.7)		0.4 (–0.7 to 1.5)		–0.5 (–1.4 to 0.3)		–0.1 (–0.9 to 0.6)		–0.1 (–0.9 to 0.8)
PN exposure		0.0 (–0.4 to 0.4)		0.4 (–0.1 to 0.9)		–0.8 (–2.0 to 0.4)		–1.5 (–2.5 to –0.5)^#^		0.0 (–0.9 to 0.9)		0.2 (–0.9 to 1.2)
PM_2.5_ dose		–0.1 (–0.4 to 0.1)		0.3 (0.0 to 0.6)*		–0.2 (–1.0 to 0.6)		0.3 (–0.3 to 0.9)		0.1 (–0.5 to 0.6)		0.1 (–0.6 to 0.8)
PM_2.5_ exposure		–0.3 (–0.7 to 0.0)**		0.0 (–0.4 to 0.4)		0.0 (–1.0 to 1.1)		0.4 (–0.5 to 1.2)		–0.1 (–0.8 to 0.7)		–0.1 (–1.0 to 0.8)
PM_10_ dose		–0.2 (–0.5 to 0.2)		0.3 (–0.2 to 0.7)		0.4 (–0.8 to 1.5)		0.1 (–0.8 to 1.0)		–0.7 (–1.5 to 0.2)		0.1 (–0.8 to 1.0)
PM_10_ exposure		–0.3 (–0.5 to 0.0)*		0.1 (–0.3 to 0.4)		0.1 (–0.8 to 1.0)		0.0 (–0.7,0.7)		–0.6 (–1.2 to 0.1)*		–0.1 (–0.8 to 0.7)
Soot dose (EC)		–0.1 (–0.3 to 0.2)		0.1 (–0.2 to 0.4)		0.2 (–0.6 to 1.0)		–0.2 (–0.8 to 0.5)		–0.2 (–0.8 to 0.3)		0.2 (–0.5 to 0.9)
Soot exposure		–0.1 (–0.4 to 0.2)		0.0 (–0.4 to 0.3)		0.1 (–0.8 to 1.0)		–0.3 (–1.0 to 0.4)		–0.3 (–0.9 to 0.4)		0.5 (–0.2 to 1.3)
Change was calculated as the estimate divided by the mean baseline value. Estimates were calculated using mixed-model analyses per IQR change (95% CI). IQRs of 2-hr mean values were as follows: PN. 18,195 pt/cm³ (exposure) and 2.40 × 10^10^ pt/m^2^ (dose); PM_2.5_, 68.1 µg/m³ (exposure) and 61.9 µg/m² (dose); PM_10_, 20.8 µg/m³ (exposure) and 32.4 µg/m² (dose); soot, 3.51 × 10^–5^/m (exposure) and 6.31 µg/m² (EC dose, calculated from soot absorption). Doses were adjusted for BSA. Exposures were adjusted for relative humidity, temperature, season, time test was taken, ambient NO_2_, cycling, and time privately spent in traffic before 0800 and between 1000 and 1600 hours. *p < 0.10. **p < 0.05. ^#^*p* < 0.01.												

*Associations between air pollution and blood coagulation markers.* We found associations between IQR increases in mean PM_2.5_ and PM_10_ exposure and a decrease in APTT (Table 4). However, PT tended to increase with increasing air pollution exposures. PN exposure was associated with a significant 1.5% decrease in factor VII, contrary to the hypothesized direction. We found no associations of air pollution with vWF, platelets, and fibrinogen. Stratified analyses showed that in car/bus trips, PM_2.5_ and PM_10_ were associated with a shorter APTT, but in cyclists they were associated with longer APTT, which was not consistent with expectations. For example, associations with APTT in cyclists with PM_2.5_ and PM_10_ exposure were 1.1% (0.2 to 2.0; *p* = 0.01) and 0.9% (0.1 to 1.7; *p* = 0.03), respectively, whereas in cars they were –0.6% (–1.0 to –0.2; *p* < 0.01) and –0.4% (–0.7 to –0.1; *p* = 0.02) [see Supplemental Material, Table 6, (http://dx.doi.org/10.1289/ehp.1003151)].

*Additional analyses.* Associations with cell counts, blood coagulation, and inflammation markers were similar when including ambient PM_10_ or O_3_ in the model instead of NO_2_ (data not shown). An analysis of the postexposure measurements instead of the post exposure – preexposure differences also did not show any consistent associations between in-traffic exposure and blood markers (data not shown). We found no significant differences after stratification into high or low BMI or high or low fruit and vitamin intake groups (data not shown). Associations between IQR increases in PN and the outcomes were similar when median and 95th percentile values were analyzed instead of mean values (data not shown).

## Discussion

Air pollution exposure during commuting was not associated with consistent changes in inflammation markers, blood cell counts, or coagulation markers in this study.

Several biological mechanisms have been proposed for the short-term effect of air pollution on cardiovascular events (Brook et al. 2010). One of the mechanisms involves airway inflammation and oxidative stress resulting in systemic inflammation, indicated by increases in cytokine levels and increases in neutrophils and other white blood cells. A second mechanism involves translocation of particles directly in the blood, resulting in systemic inflammation. Systemic inflammation may result in an acute-phase response indicated by increases in CRP and fibrinogen. Inflammation may also directly increase coagulation and thrombosis, as well as endothelial function. A third mechanism involves effects on the autonomic nervous system reflected in changes in, for example, heart rate variability. In the present study we assessed systemic inflammation including cytokines and white blood cell counts and the further potential consequences of (especially) inflammation for markers of coagulation and thrombosis.

*Blood inflammation and lung epithelium.* We found no consistent associations between air pollution exposures and inhaled doses and the inflammation markers IL6, IL8, IL10, TNFα, and CRP and the lung epithelium marker CC16 6 hr after exposure. The single study on in-traffic exposures that evaluated systemic inflammation markers found associations between in-vehicle exposure and blood levels of CRP 14 hr after exposure (Riediker et al. 2004). Controlled exposure studies of acute effects of 1- to 2-hr exposure to diesel exhaust did not find acute effects on inflammation markers (Carlsten et al. 2007; Lucking et al. 2008; Mills et al. 2007; Nightingale et al. 2000) or CC16 (Bräuner et al. 2009). The relatively large number of samples with undetectable IL6, IL8, IL10, and TNFα reduced the power of the analyses for these markers, as is evident from the larger CIs for the inflammation markers compared with the other biomarkers.

*Blood cell counts.* Although overall there was little association with blood cell counts, exposure in traffic was associated with a small decrease in leukocytes and neutrophils, contrary to the hypothesized direction. Two previous studies found increases in neutrophils 6 hr after 1-hr exposure to diesel exhaust (Salvi et al. 1999) or 14 hr after 9-hr in-vehicle exposure (Riediker et al. 2004). Jacobs et al. (2010b) found an increase in neutrophils as a percentage of leukocytes after 30-min exposure during cycling in traffic but no changes in absolute counts of neutrophils or leukocytes. Most studies did not find evidence of effects on leukocytes or neutrophils after exposures to diesel exhaust (Larsson et al. 2007; Lucking et al. 2008; Mills et al. 2005, 2007), concentrated ambient air (Gong et al. 2003), concentrated carbon particles (Routledge et al. 2006), or ultrafine particles (Gong et al. 2008). Two studies found a decrease in leukocytes after ambient exposure to PN and PM_2.5_ (Rückerl et al. 2007b) and exposure to concentrated ambient air (Ghio et al. 2003). The small decrease in neutrophils and leukocytes in the present study could be a chance finding. Alternatively, a decrease could be explained by neutrophils moving out of the bloodstream into the stressed tissue (e.g., the lungs).

*Blood coagulation markers.* We found inverse associations between PM_10_ and PM_2.5_ dose and APTT, consistent with expectations, but the positive associations observed between PT and air pollution were contrary to our hypothesis. The discrepancy between the APTT and PT findings is biologically difficult to explain. The lack of association with other markers further argues against a causal interpretation of the associations. Some studies have reported associations between air pollution exposures and vWF, fibrinogen, and platelets (Ghio et al. 2000; Mills et al. 2008; Riediker et al. 2004; Salvi et al. 1999). Other previous studies reported no associations between exposures and platelets, fibrinogen, vWF, and factor VII (Blomberg et al. 2005; Bräuner et al. 2008; Carlsten et al. 2007; Gong et al. 2003, 2008; Lucking et al. 2008; Mills et al. 2005, 2007, 2008; Routledge et al. 2006; Samet et al. 2009). The inverse association between PN exposure and factor VII may be due to chance, but it is supported by findings of Rückerl et al. (2006) and Seaton et al. (1999), who found decreases in factor VII with a 1- to 3-day lag in PN and PM_10_ exposure. Lucking et al. (2008) and Jacobs et al. (2010a) found associations with thrombus formation and platelet function 2 and 6 hr after exposure, respectively. These end points may be more specific for determination of acute coagulatory effects (Brook et al. 2010).

*Sensitivity and subgroup analyses.* The mean post exposure – preexposure exposure difference was different for cycling trips and car/bus trips for leukocytes, neutrophils, APTT, PT, IL10, and TNFα. This could be a result of the higher level of exercise during cycling. Strenuous exercise acutely affects white cell count (Kasapis and Thompson 2005), coagulation markers (Thrall et al. 2007), and inflammation markers (Febbraio and Pedersen 2002; Kasapis and Thompson 2005); moderate exercise during cycling may also influence these markers. Because we adjusted for trip type and because exercise levels were constant from day to day, these two factors have not affected the observed associations between air pollution and these end points. In a previous article describing respiratory end points in this population (Zuurbier et al. 2011), we reported small associations of PN and soot with peak flow and airway resistance, but not with exhaled NO, a marker of airway inflammation. However, we did report a positive association with exhaled NO in the car/bus trips (Zuurbier et al. 2011). In line with this, in the present study, the marker of systemic inflammation IL10 was not associated with air pollutants in the full population, but there was a positive but nonsignificant association with IL10 and soot exposure (*p* = 0.08) and soot dose (*p* = 0.08) in car/bus trips.

In the present study, we dealt with the influence of other factors, such as age, sex, obesity, and diet, on associations between air pollution and blood markers by looking at changes in markers between postexposure and preexposure values and by including random intercepts for subjects. We eliminated effects of circadian variation in blood markers by drawing the blood samples at the same time on each test day.

*Strengths and limitations.* In-vehicle studies provide a good opportunity to study associations between short-term air pollution exposure and acute health effects (Adar and Kaufman 2007). Studies of ambient air pollution face problems in exposure misclassification, but we obtained precise information on exposure of the participants. We corrected for the influence of prior ambient exposure by adjusting for levels of ambient air pollution of the day before. In controlled exposure studies, subjects are exposed to a mixture of pollutants that may be less representative of outdoor situations. We studied real-life exposure, on a route and in modes of transport that are representative of Dutch commutes. Our study included a large number of observations, leading to precise effect estimates, especially for coagulation markers and blood cell counts. The large number of exposure days resulted in a wide range in air pollution levels, sufficient to study the relation between air pollution and health effects. Correlations between air pollutant exposures were low to moderate, facilitating analyses of the separate effects of PN and PM_10_ on health outcomes. PN exposures during cycling were characterized by many high, short peaks, whereas exposures during car/bus trips were characterized by fewer and lower peaks of longer duration. There was no indication that associations with 95th percentile values were stronger than with mean or median values (data not shown). Inhalation rates were estimated using heart rates, giving valuable information on inhaled doses that were of specific importance because of the comparison of cycling trips with car/bus trips. We did not find stronger associations for doses compared with exposures.

We may not have been able to observe associations because of the study design, study population, exposure issues, or timing of the health measurements. Our study derived exposure contrast from day-to-day variation in air pollution and differences between modes of transport. Other studies have successfully used a control exposure using filtered air in a laboratory setting (Jacobs et al. 2010b), with the disadvantage that other factors in a laboratory setting (e.g., temperature, relative humidity) also differ from ambient air exposures. PN within our study ranged from a few thousand to 80,000 particles/cm^3^, which is larger than the range reported by Jacobs et al. (maximum PN 40,000 particles/cm³ versus close to zero in the laboratory). Using masks with filters as controlled exposure was not feasible during bus and cycle trips.

Our study population consisted of healthy, middle-aged adults. Effects on cardiovascular parameters have been found to be more pronounced in susceptible subgroups such as subjects with cardiovascular disease, obesity or high BMI, diabetes, and elderly (Brook et al. 2010; Dubowsky et al. 2006; Hertel et al. 2010; Rückerl et al. 2007a). Associations in our study were not different for subjects with high BMI than for subjects with low BMI (above or below 25 kg/m²) or for subjects with high or low fruit and vitamin intake.

We measured blood markers only 6 hr after exposure; therefore, we may have missed immediate and more delayed responses. There is evidence from previous studies (Gong et al. 2008; Lucking et al. 2008; Mills et al. 2008; Salvi et al. 1999) that neutrophils, CRP, and platelets may be affected within 6 hr after exposure. Concurrent-day ambient exposure was associated with increases in CRP (Peters et al. 2001b; Pope et al. 2004) and fibrinogen (Pekkanen et al. 2000), but these studies do not allow more detailed definition of time windows of response. Several controlled exposure studies have reported airway inflammation at 4–6 hr after exposure (Salvi et al. 1999; Stenfors et al. 2004), which may lead to systemic inflammation (Brook et al. 2010).

We conclude that air pollution exposure during commuting was not consistently associated with acute changes in inflammation markers, blood cell counts, or blood coagulation markers.

## Supplemental Material

(288 KB) PDF
