# Supplementary material for: In-Traffic Air Pollution Exposure and CC16, Blood Coagulation, and Inflammation Markers in Healthy Adults
Source: Environ Health Perspect. 2011 Jun 10;119(10):1384–9. doi: 10.1289/ehp.1003151 (PMC3230432; doi:10.1289/ehp.1003151)
Supplement: (288 KB) PDF [file ehp.1003151.s001.pdf]

Supplemental Material

In-traffic Air Pollution Exposure and CC16, Blood Coagulation and Inflammation Markers in Healthy Adults

Moniek Zuurbier, Gerard Hoek, Marieke Oldenwening, Kees Meliefste, Esmeralda Krop, Peter van den Hazel, Bert Brunekreef

Environmental Health Perspectives

In the supplemental material additional information is provided on exposure measurements. The supplement also presents additional information on quality of clinical measurements and additional analyses on the associations between exposure to and inhaled doses of air pollutants and changes in biomarkers.

Table of Contents

1. Additional information on exposure measurements 2
2. Details clinical measurements 3
   Table 1. *Distribution of blood markers at baseline (8 am) and mean post-pre outcome differences*
   Table 2. CV and LOD of IL6, IL8, IL10 and TNFα *4*
3. Additional analyses on associations between exposures, doses and changes in biomarkers 5
   Table 3. Effect estimates for differences in IL10 and TNFα per IQR change In exposure and inhaled dose, stratified in bicycle trips and car/bus trips 5
   Table 4. Effect estimates for differences in basophils, eosinophils, and monophils per IQR change in exposure and inhaled dose 5
   Table 5. Effect estimates for differences in neutrophils and leukocytes per IQR change in exposure and inhaled dose, stratified in bicycle trips and car/bus trips *6*
   Table 6. Effect estimates for differences in APTT and PT per IQR change in exposure and inhaled dose, stratified in bicycle trips and car/bus trips *6*
4. References 6

1. Additional information on exposure measurements

As part of QA/QC we monitored PN indoors (co-location of the three units) in one of the office buildings. Concentrations were much lower than measured outdoors in traffic. There was hence a good contrast between the two-hour commuting exposure and the prior and post-exposures.

For the real-time measurements of PN we used not only the mean, but also the median and 95th percentile (P95) of the individual readings to study the effects of peaks in exposures. The Spearman rank correlation coefficient between PN mean and median was 0.91, between PN mean and P95, 0.71, and between PN median and P95 the correlation was 0.53. For $PM_{2.5}$ we only analysed mean values, as median, P95 and P99 values were highly correlated (>0.90).

We obtained hourly ambient NO_2 and ozone data, measured with chemiluminescence, ultraviolet absorption, from the nearest urban background station (16 km distance) of the National Air Quality Monitoring Network of the Dutch National Institute for Public Health and the Environment. We obtained daily ambient PM_{10} data, measured with beta attenuation continuous monitors, from the nearest regional background station (20 km distance) as there were no urban background PM_{10} data from nearby monitoring stations available. In analyses, average ambient concentrations of the 24 hours preceding the baseline health measurements (8am) were used for NO_2 and ozone, and the mean concentration of the previous day was used for PM_{10}.

2. Details clinical measurements

Supplemental Material, Table 1. Distribution of blood markers at baseline (8 am) and mean post-pre outcome differences

	Unit	Mean baseline (SE)	Mean difference post-pre (SE)
**Inflammation markers**			
C-reactive Protein (CRP)	mg/L	1.2 (0.10)	0.00 (0.030)
Interleukine 6 (IL6)	ng/L	0.41 (0.023)	0.06** (0.018)
Interleukine 8 (IL8)	ng/L	3.0 (0.33)	-0.25* (0.12)
Interleukine 10 (IL10)	ng/L	0.37 (0.017)	-0.05** (0.012)
Tumour Necrosis Factor Alpha (TNFα)	ng/L	2.0 (0.11)	0.10 (0.11)
**Marker of lung epithelial damage**			
ClaraCell protein 16 (CC16)	µg/L	37.4 (0.83)	-11.0** (0.50)
**Blood cell counts**			
Leukocytes	10^9/L	6.5 (0.082)	0.65** (0.054)
Neutrophils	10^9/L	3.3 (0.058)	0.61** (0.050)
Basophils	10^9/L	0.03 (0.002)	0.00* (0.002)
Eosinophils	10^9/L	0.21 (0.007)	-0.06** (0.005)
Lymphocytes	10^9/L	2.4 (0.042)	0.05* (0.022)
Monocytes	10^9/L	0.52 (0.009)	0.03** (0.007)
Erythrocytes	10^{12}/L	4.9 (0.021)	-0.11** (0.008)
**Coagulation markers**			
Activated Partial Thromboplastin Time (APTT)	sec	28.2 (0.12)	-0.26** (0.040)
Prothrombine time (PT)	sec	12.6 (0.035)	-0.08** (0.23)
VonWillebrand Factor	%	107 (1.9)	-4.5** (0.52)
Factor VII	%	90.5 (0.94)	-2.4** (0.33)
Fibrinogen	g/L	3.0 (0.035)	-0.05** (0.011)
Thrombocytes	10^9/L	244 (3.2)	-2.4** (0.78)

Based on 326 to 344 observations
*) Difference post-pre exposure significant ($p<0.05$), paired t-test
**) Difference post-pre exposure significant ($p<0.01$) , paired t-test

The coefficient of variance (CV) of the CRP assay was below 1.0%. Information on CV values and Limits of Detection (LOD) of IL6, IL8, IL10 and TNFα are presented in Table 2. LODs were calculated for each two plates separately, as for the Luminex assay two plates were analysed together, with one calibration curve. For samples below the LOD we used two third of LOD. We performed 10% of the CC16 analyses in duplicate, the average CV was 11%. Average CV values of blood cell counts were 1 to 5%. Average CV values of the coagulation markers Prothrombin Time, Activated Partial Thromboplastin Time, platelets, fibrinogen, factor VII (FVII) and Von Willebrand Factor were below 2 to 7%.

Supplemental Material, Table 2. CV and LOD of IL6, IL8, IL10 and TNFα

	**Average CV (%)**	**Average LOD (range)**	**% of samples below LOD**	**% of samples at lower end of calibration curve[a]**
**IL6**	33	0.08 (0.00-0.41)	41	44
**IL8**	21	0.10 (0.02-0.29)	22	26
**IL10**	17	0.13 (0.01-0.24)	9	88
**TNFα**	35	0.38 (0.01-1.15)	33	32

[a]**)** calculated using automatic extrapolation by the Bio-plex software (version 4.2, Bio-rad Laboratories, Hercules, CA, USA).

3. Additional analyses on associations between exposures, doses and changes in biomarkers

Supplemental Material, Table 3. Effect estimates for differences in IL10 and TNFα per IQR change in exposure and inhaled dose, stratified in bicycle trips and car/bus trips

	Bicycle trips		Car and bus trips	
	**IL10 (%)**	**TNFα (%)**	**IL10 (%)**	**TNFα (%)**
**PN**				
**exposure**	-4.4 (-20,11)	-2.2 (-45,40)	2.8 (-5.6,11)	-7.8 (-28,12)
**dose**	1.3 (-6.7,9.2)	-13 (-34,8.5)	6.0 (-6.0,18)	-18.3 (-42,5.8)
**$PM_{2.5}$**				
**exposure**	-14* (-31,2.2)	-16 (-63,31)	-1.8 (-8.2,4.5)	-6.8 (-22,8.4)
**dose**	-4.7 (-13,3.6)	-18 (-41,5.6)	-0.6 (-6.4,5.2)	-6.2 (-20,7.9)
**PM_{10}**				
**exposure**	7.2 (-7.6,22)	9.0 (-32,50)	1.1 (-3.8,6.1)	-3.1 (-15,8.4)
**dose**	6.8 (-4.3,18)	-11 (-42,19)	2.7 (-4.5,9.8)	-6.5 (-22,9.4)
**Soot**				
**exposure**	-0.6 (-16,14)	-9.1 (-50,32)	5.0* (-0.5,11)	-5.9 (-19,7.3)
**dose (EC)**	2.2 (-5.6,10)	-12 (-33,8.9)	5.4* (-0.6,12)	-8.6 (-22,5.1)

Log-transformed values were used. Estimates calculated using mixed model analyses, per IQR change (95% confidence interval in parentheses). IQRs of two hour mean values: PN 18,195 pt/cm³ (exposure) and 2.40×10^{10} pt/m² (dose), $PM_{2.5}$ 68.1 µg/m³ (exposure) and 61.9 µg/m² (dose), PM_{10} 20.8 µg/m³ (exposure) and 32.4 µg/m² (dose), soot 3.51×10^{-5}/m (exposure) and 6.31 µg/m² (EC dose, calculated from soot absorption). Doses are adjusted for body surface area. Adjusted for RH, temperature, season, time test was taken, ambient NO_2, cycling, and time privately spent in traffic before 8am and between 10am and 4pm.
*) p<0.10
**) p<0.05
***) p<0.01

Supplemental Material, Table 4. Effect estimates for differences in basophils, eosinophils, and monophils per IQR change in exposure and inhaled dose

	**Basophils (%)**	**Eosinophils (%)**	**Monophils (%)**
**PN**			
**exposure**	-20 (-60,20	0.5 (-2.4,3.3)	1.5 (-2.5,5.6)
**dose**	-13 (-47,23)	-0.5 (-2.4,1.9)	1.7 (-1.7,5.2)
**$PM_{2.5}$**			
**exposure**	-6.7 (-43,27)	1.0 (-1.4,3.3)	1.7 (-1.7,5.2)
**dose**	-3.3 (-30,20)	0.5 (-1.0,2.4)	1.7 (-0.8,4.2)
**PM_{10}**			
**exposure**	-37 (-63,-6.7)	1.4 (-0.5,3.3)	1.7 (-1.0,4.6)
**dose**	-33 (-73,3.3)	1.4 (-0.5,3.8)	3.5 (-0.2,7.1)
**Soot**			
**exposure**	-20 (-50,6.7)	0.0 (-1.9,1.9)	0.6 (-2.3,3.5)
**dose (EC)**	-13 (-40,13)	0.5 (-1.4,1.9)	1.2 (-1.5,3.7)

Change was calculated as estimate divided by the mean baseline value. Estimates calculated using mixed model analyses, per IQR change (95% confidence interval in parentheses). IQRs of two hour mean values: PN 18,195 pt/cm³ (exposure) and 2.40×10^{10} pt/m² (dose), $PM_{2.5}$ 68.1 µg/m³ (exposure) and 61.9 µg/m² (dose), PM_{10} 20.8 µg/m³ (exposure) and 32.4 µg/m² (dose), soot 3.51×10^{-5}/m (exposure) and 6.31 µg/m² (EC dose, calculated from soot absorption). Doses are adjusted for body surface area. Adjusted for RH, temperature, season, time test was taken, ambient NO_2, cycling, and time privately spent in traffic before 8am and between 10am and 4pm.
*) p<0.10
**) p<0.05

Supplemental Material, Table 5. Effect estimates for differences in neutrophils and leukocytes per IQR change in exposure and inhaled dose, stratified in bicycle trips and car/bus trips

	**Bicycle trips**		**Car and bus trips**	
	**Leukocytes (%)**	**Neutrophils (%)**	**Leukocytes (%)**	**Neutrophils (%)**
**PN**				
**exposure**	-4.9* (-10,0.6)	-11** (-21,-0.7)	1.0 (-1.8,3.8)	0.4 (-4.3,5.1)
**dose**	0.4 (-2.6,3.4)	-0.4 (-6.2,5.4)	0.0 (-4.1,4.0)	-3.1 (-10,4.0)
**$PM_{2.5}$**				
**exposure**	-5.8** (-12,-0.1)	-8.8 (-20,1.9)	-0.2 (-2.3,1.9)	-2.4 (-5.9,1.1)
**dose**	-2.9* (-5.8,0.0)	-4.6 (-10,0.9)	-0.2 (-2.2,1.7)	-2.6 (-5.9,0.6)
**PM_{10}**				
**exposure**	-1.3 (-6.6,4.0)	1.2 (-8.8,11)	-1.1 (-2.9,0.6)	-1.7 (-4.6,1.3)
**dose**	1.4 (-2.8,5.6)	3.9 (-4.1,12)	-1.3 (-3.8,1.1)	-2.1 (-6.4,2.2)
**Soot**				
**exposure**	-5.0* (-10,0.3)	-7.7 (-18,2.4)	-0.4 (-2.3,1.4)	-1.2 (-4.2,1.9)
**dose (EC)**	-0.4 (-3.2,2.5)	-0.7 (-6.1,4.7)	-0.9 (-2.9,1.2)	-2.2 (-5.6,1.3)

Change was calculated as estimate divided by the mean baseline value. Estimates calculated using mixed model analyses, per IQR change (95% confidence interval in parentheses). IQRs of two hour mean values: PN 18,195 pt/cm³ (exposure) and $2.40*10^{10}$ pt/m^2 (dose), $PM_{2.5}$ 68.1 µg/m³ (exposure) and 61.9 µg/m² (dose), PM_{10} 20.8 µg/m³ (exposure) and 32.4 µg/m² (dose), soot $3.51*10^{-5}$/m (exposure) and 6.31 µg/m² (EC dose, calculated from soot absorption). Doses are adjusted for body surface area. Adjusted for RH, temperature, season, time test was taken, ambient NO_2, cycling, and time privately spent in traffic before 8am and between 10am and 4pm.
*) p<0.10, **) p<0.05

Supplemental Material, Table 6. Effect estimates for differences in APTT and PT per IQR change in exposure and inhaled dose, stratified in bicycle trips and car/bus trips

	**Bicycle trips**		**Car and bus trips**	
	**APTT (%)**	**PT (%)**	**APTT (%)**	**PT (%)**
**PN**				
**exposure**	0.5 (-0.4,1.3)	-0.4 (-1.4,0.6)	-0.1 (-0.6,0.5)	0.5 (-0.1,1.2)
**dose**	0.0 (-0.4,0.5)	0.1 (-0.5,0.7)	0.0 (-0.7,0.8)	0.2 (-0.7,1.1)
**$PM_{2.5}$**				
**exposure**	1.1 (0.2,2.0)	0.7 (-0.3,1.7)	-0.6 (-1.0,-0.2)	-0.1 (-0.6,0.5)
**dose**	0.6 (0.2,1.1)	0.4 (-0.1,1.0)	-0.4 (-0.8,-0.1)	-0.1 (-0.5,0.4)
**PM_{10}**				
**exposure**	0.9 (0.1,1.7)	-0.1 (-1.1,0.9)	-0.4 (-0.7,-0.1)	0.2 (-0.3,0.6)
**dose**	0.4 (-0.3,1.0)	0.2 (-0.6,1.0)	-0.3 (-0.8,0.1)	0.2 (-0.4,0.7)
**Soot**				
**exposure**	0.6 (-0.2,1.4)	-0.4 (-1.4,0.6)	-0.3 (-0.6,0.1)	-0.1 (-0.6,0.4)
**dose (EC)**	0.2 (-0.3,0.6)	0.0 (-0.5,0.5)	-0.2 (-0.6,0.2)	-0.1 (-0.5,0.4)

Change was calculated as estimate divided by the mean baseline value. Estimates calculated using mixed model analyses, per IQR change (95% confidence interval in parentheses). IQRs of two hour mean values: PN 18,195 pt/cm³ (exposure) and $2.40*10^{10}$ pt/m^2 (dose), $PM_{2.5}$ 68.1 µg/m³ (exposure) and 61.9 µg/m² (dose), PM_{10} 20.8 µg/m³ (exposure) and 32.4 µg/m² (dose), soot $3.51*10^{-5}$/m (exposure) and 6.31 µg/m² (EC dose, calculated from soot absorption). Doses are adjusted for body surface area. Adjusted for RH, temperature, season, time test was taken, ambient NO_2, cycling, and time privately spent in traffic before 8am and between 10am and 4pm.
*) p<0.10, **) p<0.05, ***) p<0.01

4. References

Zuurbier M, Hoek G, Van den Hazel P, Brunekreef B. Minute ventilation of cyclists, car and bus passengers: an experimental study. Environ Health 8:48 (2009).
